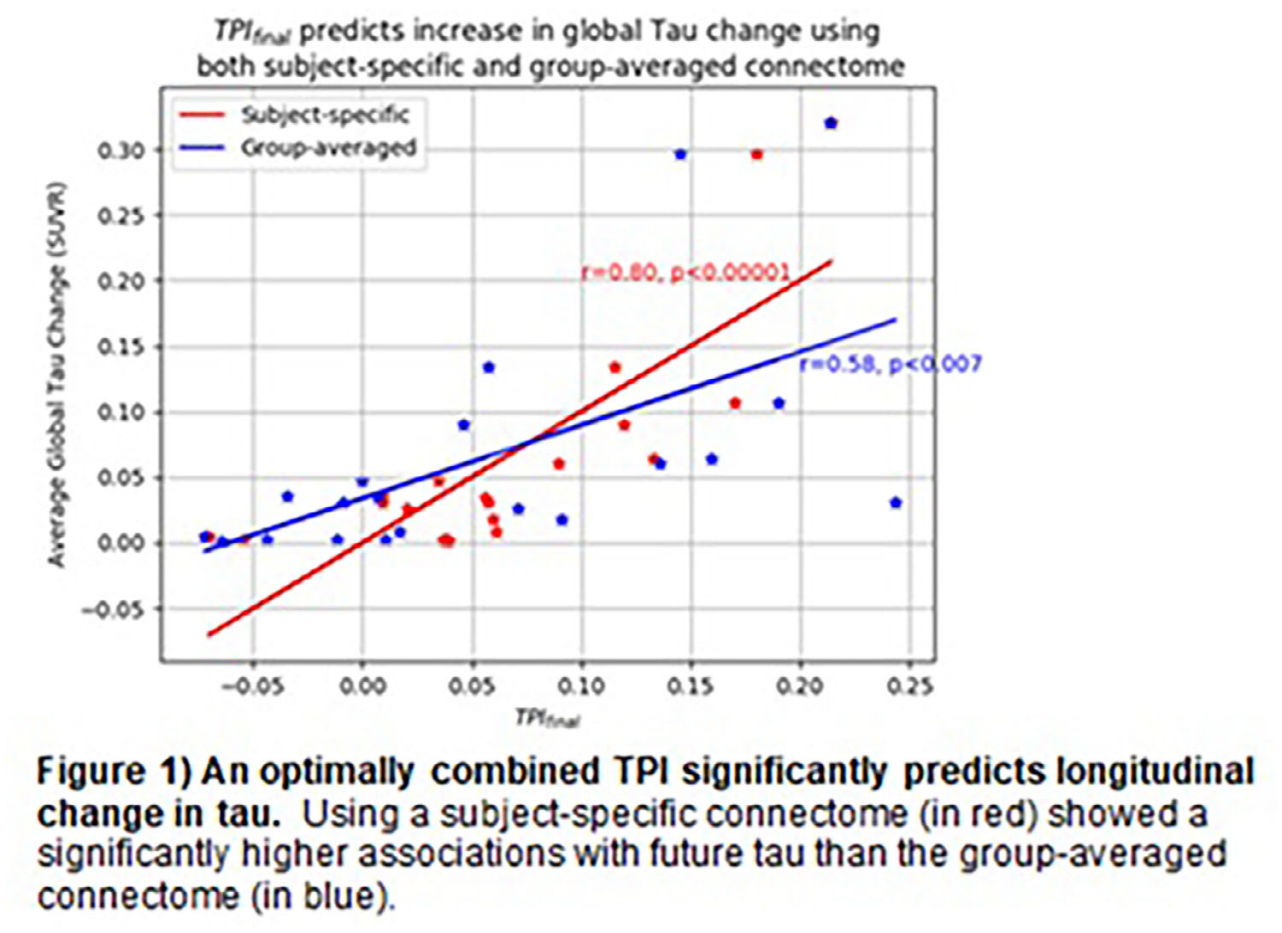# The Tau Progression Index (TPI): an individualized, clinically applicable, multimodally‐derived score to predict AD progression

**DOI:** 10.1002/alz70856_101039

**Published:** 2025-12-25

**Authors:** Gloria Chiang, Seyed Hani Hojjati, Bardiya Ghaderi Yazdi, Sindy Ozoria, Peter Chernek, Jenseric Calimag, Yaakov Stern, Davangere P. Devanand, Sonja Blum, Tracy A Butler, Silky Pahlajani, Christian G Habeck, Hengda G He, Nancy Foldi, Mohammad Khalafi, Qolamreza R Razlighi

**Affiliations:** ^1^ Weill Cornell Medicine, New York City, NY, USA; ^2^ Taub Institute for Research in Alzheimer's Disease and the Aging Brain, Columbia University, New York, NY, USA; ^3^ Columbia University Vagelos College of Physicians and Surgeons, New York, NY, USA; ^4^ Columbia University Irving Medical Center, New York, NY, USA; ^5^ Taub Institute for Research on Alzheimer's Disease and the Aging Brain, Columbia University, New York City, NY, USA

## Abstract

**Background:**

Alzheimer's Disease (AD) prognosis is extremely heterogeneous, even with a similar burden of global tau and amyloid (Aβ) deposition in the brain, which makes it challenging to develop targeted therapeutic interventions and to counsel families on disease prognosis.

**Method:**

The TPI is based on 4 components: 1) Remote interaction (TPIri) between Aβ and tau pathologies in regions that are functionally and/or structurally connected, but spatially distinct; 2) Local interaction (TPIli) between spatially co‐localized Aβ and tau pathology; and subject‐specific 3) Functional connectivity (TPIfc) and 4) Structural connectivity (TPIsc) between regions that can facilitate the spread of tau in the brain.

**Result:**

From an ongoing study of 112 participants with early accumulation of Ab and Tau, longitudinal data were available on 27 participants (2‐3 years of follow‐up). Using these data and all 4 TPI components as independent variables, we built a LASSO model to predict future tau accumulation, controlling for covariates. The obtained coefficients were used to compute our TPI, and its predictability was assessed by its association with actual longitudinal tau accumulation (within‐sample validation). Furthermore, we compared our model, using subject‐specific connectomes, with a conventional model using group‐averaged connectomes. As seen in Figure 1, while both TPIs (obtained by subject‐specific; r=0.8, *p* <10‐5; and group‐averaged connectomes: r=0.58, *p* <0.007) predicted longitudinal tau accumulation, the subject‐specific TPI significantly outperformed the group‐averaged TPI in predicting subsequent tau (DSlope t=2.96, *p* <0.009) and accounts for 30% more variance in the prediction.

**Conclusion:**

Despite a small sample size, we demonstrate that an imaging index that incorporates baseline Ab, tau, and subject‐specific connectivity can accurately predict future accumulation of tau. Validation in a larger cohort is ongoing.